# Inhibition of Aflatoxin B1 Production by Procyanidins Present in *Annona muricata* and *Uncaria tomentosa* Aqueous Extracts

**DOI:** 10.3390/toxins16110454

**Published:** 2024-10-23

**Authors:** Laura F. Cadenillas, Guillaume Billerach, Christopher Hernandez, Vanessa Durrieu, Jean-Denis Bailly

**Affiliations:** 1 Laboratoire de Chimie Agro-Industrielle (LCA), Université de Toulouse, INRAE, INPT, 4 Allée Emile Monso, 31030 Toulouse, France; laura.cadenillas.s@gmail.com (L.F.C.); guillaume.billerach@toulouse-inp.fr (G.B.); hernandezhernandezchristopher@gmail.com (C.H.); vanessa.durrieu@ensiacet.fr (V.D.); 2 École Nationale Vétérinaire de Toulouse, 23 Chemin des Capelles, CEDEX, 31076 Toulouse, France

**Keywords:** Aflatoxin B_1_, *Annona muricata*, *Uncaria tomentosa*, antioxidant activity, procyanidins

## Abstract

Aflatoxin B_1_ (AFB_1_), primarily produced by *Aspergillus flavus* and *A. parasiticus*, is the most dangerous mycotoxin for humans and contaminates a variety of crops. To limit fungal growth and aflatoxin production in food and feed, research has been increasingly focusing on alternatives to pesticides. Studies show that some aqueous plant extracts with strong antioxidant properties could significantly impact AFB_1_ production, representing an eco-friendly and sustainable method to protect crops. The present study demonstrates that aqueous extracts of *Anonna muricata* (*AM*) and *Uncaria tomentosa* (*UT*) inhibit AFB_1_ synthesis in a dose-dependent manner with a half-maximal inhibitory concentration of 0.25 and 0.28 mg dry matter per milliliter of culture medium, respectively. This effect correlates with the presence of polyphenols and, more precisely, with condensed tannins. It is also related to the subsequent antioxidant activity of both extracts. A bio-guided fractionation followed by high-performance liquid chromatography and mass spectrometry analysis of the active fractions identifies procyanidins and, more precisely, catechin (5.3% *w*/*w* for *AM* and 5.4% *w*/*w* for *UT*) and epicatechin (10.6% *w*/*w* for *AM* and 25.7% *w*/*w* for *UT*) as the major components in both extracts. The analysis of how pure standards of these molecules affect AFB_1_ production demonstrates that catechin plays an essential role in the inhibition observed for both plant extracts, since the pure standard inhibits 45% of AFB_1_ synthesis at a concentration close to that of the extracts.

## 1. Introduction

Aflatoxin B_1_ (AFB_1_) is a mycotoxin produced by several fungal species within the section *Flavi* of the genus *Aspergillus*. *Aspergillus flavus* and *A. parasiticus* are the two major aflatoxin-producing species and are consistently responsible for the contamination of various types of foods worldwide. AFB_1_ is the most dangerous mycotoxin and is classified as a class 1 carcinogen for humans by the International Agency for Research on Cancer. It is responsible for hepatocellular carcinoma in humans and has other toxic effects such as growth suppression, immune system modulation, and malnutrition [1].

AFB_1_ poses a major risk to public health, particularly in warm regions where environmental conditions favor the growth of toxigenic fungi. Aflatoxigenic species can grow on various foods such as cereals, groundnuts, and spices. Due to the physiological characteristics of producing species and their xerophilic character, AFB_1_ can enter at different steps of the production chain: in the fields before harvest, during the peri-harvest period, before crops are sufficiently dried or later, during storage in the case of insufficient drying or remoistening. Given the stability of AFB_1_, the most effective strategy to protect consumers is to limit the toxin synthesis and the subsequent contamination of food.

Various strategies have now been established to limit or prevent fungal development and subsequent AFB_1_ synthesis in crops during pre-harvest, post-harvest, and storage of foods. Some of these strategies typically include good agricultural practices, although the key role of climate, which cannot be controlled, may strongly limit their efficacy. In addition, fungicides are used mainly to avoid the growth of toxigenic fungi, but their numerous harmful side effects and the increasing resistance of target organisms require alternative strategies to prevent aflatoxin contamination of crops [2,3].

Ensuring food safety for consumers while developing sustainable production tools with minimal environmental impact has triggered extensive studies of new eco-friendly approaches based on natural products, such as plant-derived substances. Such products could be used to limit contamination in the fields, during peri-harvest by coating grains, and even during storage, although it would require specific formulations to guarantee the homogenous distribution of the product in silos.

Various plant-derived molecules limit the synthesis of mycotoxins such as AFB_1_ [4,5,6,7,8,9]. For example, polyphenols such as phenols, flavonoids, tannins, and terpenes are active molecules that inhibit fungal development and AFB_1_ production [10,11,12]. Additionally, certain active molecules with antioxidant potential may inhibit AFB_1_ production by regulating the oxidative stress around the fungus [13].

Current research suggests that condensed tannins, present in some plant extracts, may significantly inhibit some mycotoxins. For example, a study on the condensed tannins of *Dalea purpurea* reported that these compounds restrict the synthesis of deoxynivalenol and ochratoxin A [14]. Similarly, another study on the aqueous extract of *Mimosa tenuiflora* demonstrated that condensed tannins inhibit AFB_1_ synthesis due to a down-regulation of the internal regulators (*aflR* and *aflS*) of AFB_1_ cluster genes [15].

The leaves of the *Annona muricata* (*AM*) tree are widely used in infusions to treat diabetes, insomnia, cystitis, and rheumatic problems [16,17,18]. To date, a total of 212 bioactive compounds have been reported in *AM* leaves, the predominant ones being acetogenins, alkaloids, and polyphenols [19,20].

In the same way, *Uncaria tomentosa* (*UT*), a large woody vine native to the Amazon, has antioxidant, antimicrobial, and anti-inflammatory properties and can also be used to treat asthma, abscesses, and urinary tract infections [21,22,23]. More than 50 phytochemicals from the plant have been identified and isolated, with condensed tannins being the most abundant. Others include indole and oxindole alkaloids, quinic acid, and polyphenols [24,25].

Previous work demonstrated that *AM* and *UT* aqueous extracts are rich in condensed tannins, which contribute to the high antioxidant potential of these plants [6]. Considering the antiradical capacity of these plant extracts and the potential link between AFB_1_ synthesis and oxidative stress levels, the present study seeks to determine whether polyphenols, and more precisely condensed tannins, present in the aqueous extracts of *AM* and *UT* are responsible for the anti-AFB_1_ activity. We thus used bio-guided fractionation of the extracts followed by high-performance liquid chromatography (HPLC) and mass spectrometry analysis of the fractions to identify the condensed tannins responsible for AFB_1_ inhibition. Additionally, we evaluated how the major condensed tannins present in extracts affect the inhibition of AFB_1_.

## 2. Results and Discussion

### 2.1. Effect of AM and UT Aqueous Extracts on A. flavus Growth and AFB_1_ Synthesis

**Figure 1:**
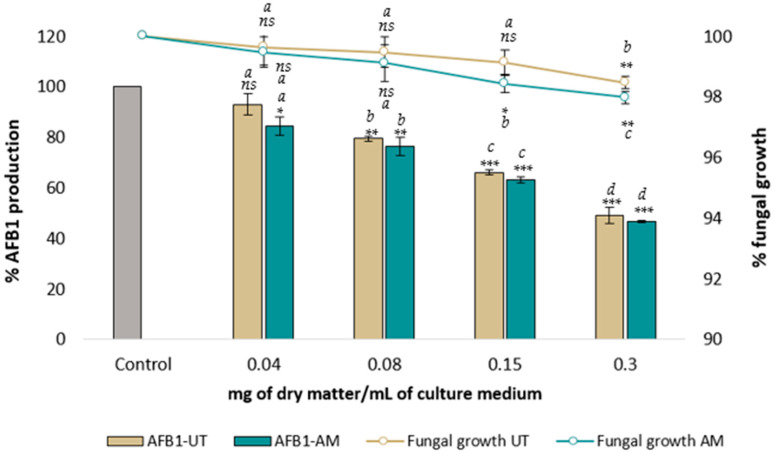
Dose effect of increasing concentrations of *UT* (brown) and *AM* (blue) aqueous extract on *A. flavus* NRRL 62,477 growth (lines) and AFB_1_ production (bars). Results are presented as the percentage of AFB_1_ production or colony diameter relative to untreated control cultures ± one standard deviation (*n* = 4). Control and treated cultures with statistically significant differences are indicated by * (*ns* = no statistically significant change, * *p* value < 0.05, ** *p* value < 0.01, *** *p* value < 0.001). Significant differences between two successive concentrations of the same sample are denoted by different letters (*p* value < 0.05).

The impacts of four increasing concentrations (from 0.04 to 0.30 mg of dry matter per milliliter of culture medium) of aqueous extracts of *AM* leaves and *UT* bark on both fungal growth and AFB_1_ synthesis were evaluated after one week of incubation at 27 °C and compared with a control culture grown on an extract-free culture medium. Figure 1 shows the results.

The aqueous extracts of *AM* and *UT* did not strongly affect the growth of *A. flavus*. A slight reduction in growth occurred for concentrations of 0.15 and 0.3 mg dry matter per milliliter (DM/mL) for *AM* and 0.3 mg DM/mL for *UT*. At the highest extract concentrations, the maximal inhibition of growth was only 3% and 2.5% for *AM* and *UT*, respectively.

Both *AM* and *UT* aqueous extracts led to a very similar dose-dependent inhibition of AFB_1_ synthesis, with half-maximal inhibitory concentrations on AFB_1_ synthesis (IC50*_AFB_*_1_) of 0.25 and 0.28 mg DM/mL, respectively. The maximal observed inhibitions were 53% for *AM* and 51% for *UT*, with both occurring at 0.3 mg DM/mL culture medium.

*AM* aqueous extract reduced AFB_1_ production more than reported for other plants of the same family (*Annonaceae*). For example, *Polyalthea longifolia* and *Artabotrys odoratissimus* have IC50*_AFB_*_1_ values of 0.9 and 0.5 mg DM/mL, respectively [26,27]. The results for *UT* were in the same range as those obtained in previous studies on bark extracts of other plants. For example, *Sapindus mucorossi* and *Mimosa tenuiflora* have an IC50*_AFB_*_1_ of 0.4 and 0.15 mg DM/mL, respectively [15,26].

### 2.2. Characterization and Fractionation of AM and UT Extracts

#### 2.2.1. Composition of Extracts and Fractions

A bio-guided fractionation of *AM* and *UT* extracts was performed to better characterize the role of polyphenols and, more particularly, condensed tannins in AFB_1_ inhibition. The fractionation proceeded by separating the components using macroporous absorption resin (amberlite FPX66), leading to fraction F1 (aqueous fraction) and fraction F2 (ethanolic fraction). Next, F2 was further fractionated into a tannin-free fraction F2.1 using polyvinylpolypyrrolidone (PVPP) precipitation. The initial extracts and their fractions and sub-fractions were analyzed to determine their polyphenol content, condensed tannin concentration, and antioxidant activity. Table 1 summarizes the results.

The aqueous extract of *AM* had a polyphenol content of 188 mg GAE/g DM and an antioxidant activity equivalent to an IC50_DPPH_ of 35 mg/L. To compare, Trolox and quercetin, two strong antioxidant molecules often used as standards, have IC50_DPPH_ = 3.5 mg/L [28] and 8.1 mg/L [29], respectively, confirming that *AM* has a significant antioxidant activity. Both polyphenol content and antioxidant activity exceeded those previously reported for different *AM* leaf extracts, whether they were aqueous (polyphenol content = 155 mg GAE/g and IC50_DPPH_ = 81 mg/L) [30] or methanolic (IC50_DPPH_ of 63 mg/L) [31]. Antioxidant activity also exceeded that reported for plants belonging to the same family, such as *A. diversifolia* (IC50_DPPH_ = 98 mg/L), *A. purpurea* (IC50_DPPH_ = 151 mg/L), and *A. reticulata* (IC50_DPPH_ = 126 mg/L) [32].

*UT* extract exhibited considerably greater antioxidant activity (IC50_DPPH_ = 15 mg/L) than *AM*, which could be linked to *UT*’s greater polyphenolic compound content (226 mg GAE/g DM) and, more specifically, condensed tannins (219 mg/g DM). Note that the extract was prepared from *UT* bark, which is rich in proanthocyanidins (condensed tannins), as demonstrated by Gonçalves et al. [33]. In that work, chromatographic analysis (HPLC-diode array detection and thin-layer chromatography) revealed that the main constituents of a *Uncaria tomentosa* bark decoction were proanthocyanidins and phenolic acids. In addition, the authors demonstrated that proanthocyanidins correlate directly with the high antioxidant activity of the aqueous extract.

The fractions of *AM* and *UT* showed similar trends. As expected, F1 (aqueous fraction) had a low polyphenol content (56 mg GAE/g DM for *AM* and 18 mg GAE/g DM for *UT*), which explains the low antioxidant capacity of this fraction (IC50_DPPH_ = 310 mg/L for *AM* and IC50_DPPH_ = 363 mg/L for *UT*). In contrast, F2 (ethanolic fraction) concentrated the polyphenols: 320 mg GAE/g DM for *AM* and 530 mg GAE/g DM for *UT*. Condensed tannins represented a significant fraction of these polyphenols (220 mg/g for *AM* and 502 mg/g for *UT*), as highlighted by the low residual polyphenol content after tannin precipitation with PVPP. Fraction F2 of both plants also displayed high antioxidant activities (IC50_DPPH_ of 20 and 8 mg/L for *AM* and *UT*, respectively).

These results are consistent with each other because the fractionation used solvents of different polarities to separate the compounds of interest in the extract. Fraction F1 used only water as an elution solvent, which favored the isolation of sugars and proteins. In contrast, fraction F2 was obtained using ethanol, which favored the elution of polyphenols from the column. These results are consistent with previous studies, demonstrating the high concentration of polyphenols in the ethanolic extracts of different plants [34,35]. The correlation of the polyphenol content of the plant extract with the antioxidant activity of the extract has also been shown [36,37], as in our study.

Previous reports state that condensed tannins such as procyanidin trimer C1 and dimer B1 participate strongly in the antioxidant activity of the *AM* extract [38]. Similarly, in *UT*, condensed tannins such as procyanidins are the most abundant polyphenols in the plant and are responsible for its high antioxidant activity [39]. Since both *AM* and *UT* tannin-free subfractions (F2.1) completely lost their antioxidant capacity, our results confirm the central role of condensed tannins in both *AM* and *UT* antioxidant activity.

#### 2.2.2. Impact of *AM* and *UT* Fractions on AFB_1_ Production

**Figure 2:**
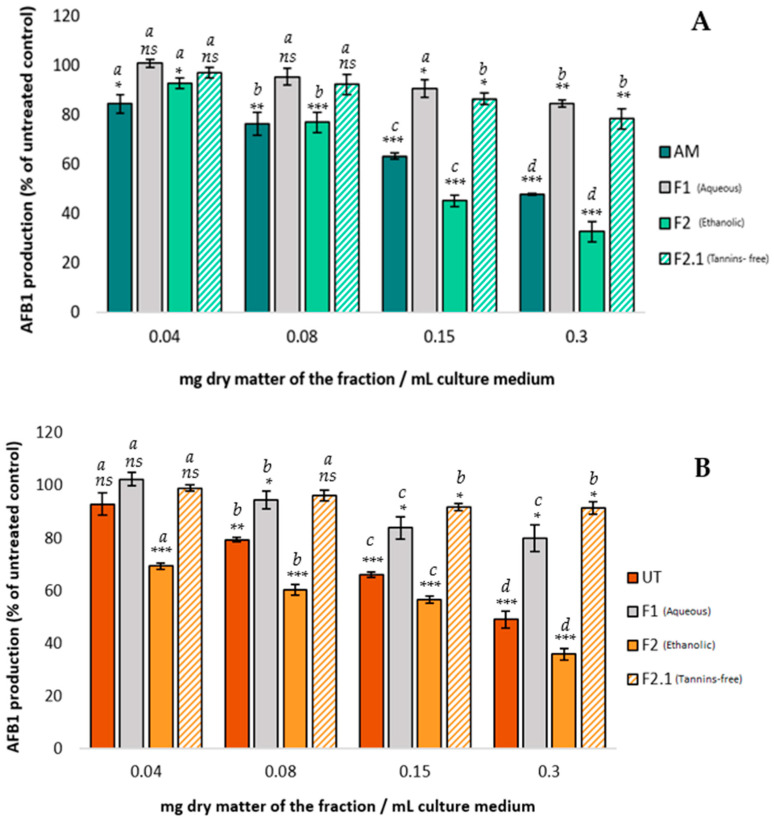
Comparison of the effect of increasing concentrations of *AM* (**A**) and *UT* (**B**) aqueous extracts and their fractions on AFB_1_ synthesis in *A. flavus* NRRL 62,477. Results are expressed as the percentage of AFB_1_ production relative to untreated control cultures ± one standard deviation (*n* = 4). Statistically significant differences from the control are indicated with * for the different concentrations of each treatment (*ns* = no statistically significant change between two concentrations, * *p* value < 0.05, ** *p* value < 0.01, *** *p* value < 0.001). Significant differences between two successive concentrations of the same extract and fraction are denoted by different letters (*p* value < 0.05).

A well-established link exists between oxidative stress and AFB_1_ synthesis. An increase in oxidative stress increases AFB_1_ synthesis, whereas a decrease, obtained by the presence of antioxidant compounds, decreases AFB_1_ production [13]. Different polyphenols with potent antioxidant capacity were reported to subsequently hinder AFB_1_ synthesis [40,41,42]. Therefore, the AFB_1_ inhibition observed with *AM* and *UT* extracts may be attributed to their high polyphenol content and the resulting significant antioxidant activity. To corroborate this claim, we tested on *A. flavus* the AFB_1_-inhibiting potential of the different fractions by using various concentrations in polyphenols, condensed tannins, and antioxidative potential. Figure 2 presents the results.

The *AM* and *UT* fractions produced similar trends in AFB_1_ inhibition.

Compared with the initial extracts, fractions F1 lost most of their ability to inhibit AFB_1_ in *A. flavus.* At the highest concentration tested (0.3 mg DM/mL), *AM* and UT aqueous fractions 1 inhibited only 11% and 20% of AFB_1_ synthesis, respectively. However, a slight bioactivity remained for both plants, which could be explained by the residual content of polyphenols found in these fractions (see Table 1). In addition, at the lowest concentrations tested (0.04 mg DM/mL), the F1 fractions of both plants induced a slight increase in AFB_1_ synthesis, possibly due to the presence of molecules such as sugars, which may supply nutritional support for the growth of *A. flavus* and, therefore, indirectly promote the synthesis of AFB_1_.

The ethanolic fractions F2 of both *AM* and *UT* produced a dose-dependent effect on AFB_1_ inhibition. The F2 fractions of both *AM* and *UT* inhibited approximately 65% of AFB_1_ synthesis at 0.3 mg DM/mL, the highest concentration tested. Since the F2 fractions were rich in polyphenolic compounds (32% *w*/*w* for *AM* and 53% *w*/*w* for *UT*), these results indicate that polyphenols may be what allows *AM* and *UT* extracts to inhibit AFB_1_ synthesis.

Removing the condensed tannins by precipitation (producing fraction F2.1) significantly reduced the efficacy of the extract, with a residual maximum AFB_1_ inhibition of 22% for *AM* and only 9% for *UT*. This result demonstrates that condensed tannins play a pivotal role in AFB_1_ inhibition for both plants.

Further studies are needed to elucidate the precise mechanisms by which condensed tannins influence AFB_1_ production. Current theories mainly propose that their antioxidant activity could be due to direct interference with the regulation of genes involved in aflatoxin biosynthesis. In fact, numerous studies have demonstrated a direct correlation between oxidative stress and the regulation of AFB_1_ synthesis [12,13,15]. Some researchers even proposed that, in *A. flavus*, aflatoxin synthesis could be part of the response to oxidative stress [43]. For example, previous work showed that extracts from the bark of *Mimosa tenuiflora* and Pine Bark, which are rich in condensed tannins, inhibit AFB_1_ synthesis by down-regulating the key regulatory genes *aflR* and *aflS* [15]. In addition, the extracts also interfere with the expression of various genes involved in the fungal stress response and antioxidant defense system (e.g., catA, sod1, mtfA, atfA, and msnA), suggesting a connection between the two processes.

Other studies suggested that extracts containing flavonoids could directly degrade AFB_1_, most likely by targeting the furan double bond and the lactone ring of AFB_1_, which are responsible for its toxic and carcinogenic properties. However, the reaction mechanisms were not fully elucidated and could involve additive or synergistic mechanisms with enzymes [12,44,45].

#### 2.2.3. Identification of the Primary Condensed Tannins in the Ethanolic Fractions of *AM* and *UT*

Research has yet to identify which type(s) of condensed tannins is (are) responsible for the anti-AFB_1_ property. Thus, we further characterized the tannins present in our extracts.

**Figure 3:**
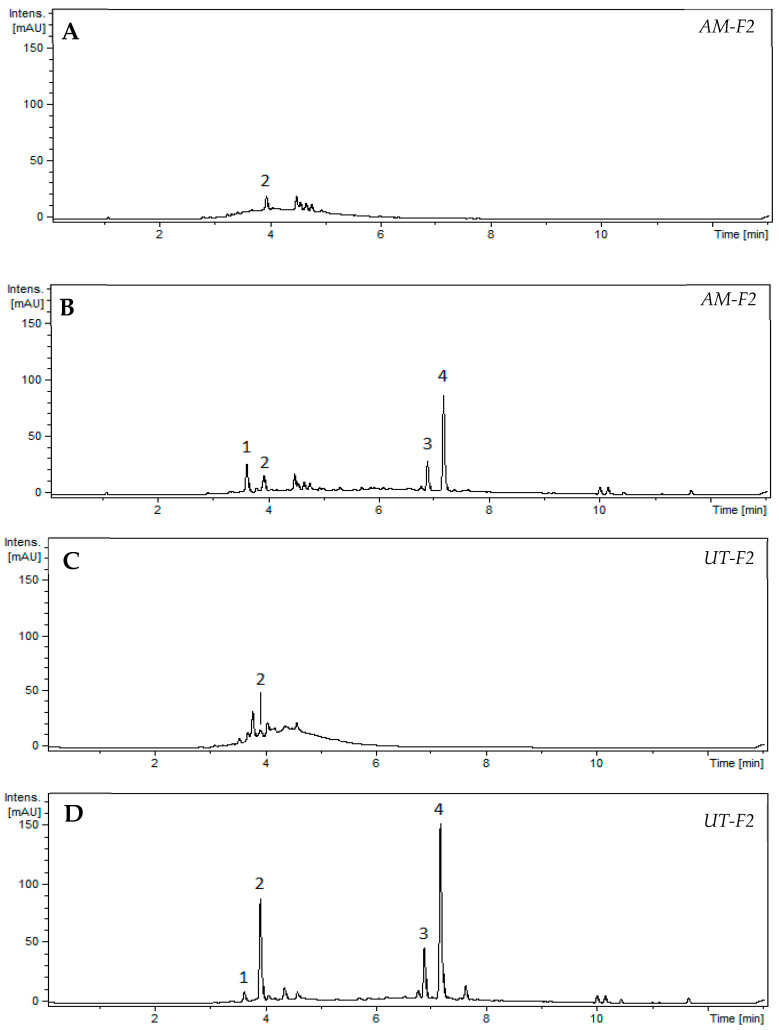
Chromatograms at 280 nm of condensed tannins present in fraction 2 of (**A**,**B**) *AM* and (**C**,**D**) *UT*, (**A**,**C**) before and (**B**,**D**) after depolymerization (1 g/L; 120 min reaction). Peak identification: **1**, Catechin (290.9 *m*/*z*); **2**, Epicatechin (290.9 *m*/*z*); **3**, Catechin-menthofuran (439.1 *m*/*z*); **4**, Epicatechin-menthofuran (439.1 *m*/*z*).

Since condensed tannins are complex polymers, their characterization required depolymerization. Therefore, condensed tannins from fractions F2 of both plants were depolymerized for 120 min in a methanolic solution of hydrochloric acid and menthofuran. Next, the samples were analyzed using ultrahigh-performance liquid chromatography (UHPLC) with ultraviolet (UV) diode-array detection (DAD) coupled with mass spectrometry (MS). Figure 3 shows the UV chromatogram of the F2 fractions at 280 nm before and after depolymerization.

Table 2 details the nature of condensed tannins (% *w*/*w*) in *AM* and *UT* F2 fractions. The results show that, in the *AM* F2 fraction, the total procyanidin content represented 15.9% (*w*/*w*) of the extract and consisted of 5.3% of catechin and 10.6% of epicatechin. In contrast, *UT* had twice the content: 31.1% (*w*/*w*) total procyanidin content from the extract, with 5.4% catechin and 25.7% epicatechin.

The difference in the percentage of condensed tannins between *AM* and *UT* fractions is primarily attributed to the part of the plant used to prepare each extract. Whereas *UT* extract was prepared from bark, *AM* extract was prepared from leaves. Recall that bark is particularly rich in condensed tannins, more so than other plant parts. This fact was illustrated by Killedar and More in a study that quantified and characterized condensed tannins from different parts of *Memecylon umbellatum*. They demonstrated that bark contains the highest concentration of condensed tannins (24.1% *w*/*w*), followed by leaves (17.6% *w*/*w*) [46].

The nature of the condensed tannins identified from *AM* and *UT* is consistent with the composition of similar extracts reported in the literature. Besides catechin and epicatechin, procyanidins B2, B4, and C1 have been identified as the primary condensed tannins in ethanolic extracts of *UT* bark [47]. Propelargonidins reported in *UT* extracts [39] were also detected in our fractions [*m*/*z* = 423.1, tentatively identified as (epi)afzelechin-menthofuran]. However, they were not quantified because of their negligible contribution to the UV chromatogram compared with procyanidins. Furthermore, a study on an ethanolic fraction of *AM* reported (+)-catechin, gallocatechin, procyanidin dimer B1, and procyanidin trimer C1 as the main flavonoids [48,49]. All these compounds except gallocatechin were detected in our fractions, but at different concentrations, which can be explained by differences in species, age, and the part of the plant collected and its geographical origin. Additionally, considering that condensed tannins are synthesized by a defense mechanism against environmental stress, their production is closely related to the external conditions and cultivation in which the plants grow [50,51].

#### 2.2.4. Effect of Catechin and Epicatechin on AFB_1_ Synthesis

Catechin and epicatechin were identified as the primary condensed tannins in *UT* and *AM* extracts. When *AM* aqueous extract was cultured with *A. flavus* at the highest concentration (0.3 mg DM/mL of culture medium), 16.2 µg of catechin and 32.1 µg of epicatechin per milliliter of culture medium were quantified, achieving an AFB_1_ inhibition of 53%. While using *UT* extract, a concentration of 15.9 µg of catechin and 77.1 µg of epicatechin per milliliter of culture medium resulted in a 51% AFB_1_ inhibition. Therefore, to determine how these compounds affect the inhibition of AFB_1_, we analyzed how AFB_1_ synthesis is affected by increasing concentrations of pure catechin and epicatechin. Specifically, we used 5, 10, and 15 µg/mL of culture medium for catechin and 6.25, 12.5, and 25 µg/mL of culture medium for epicatechin. After a seven-day incubation at 27 °C, the highest concentration of catechin (epicatechin) inhibited AFB_1_ synthesis by 45% (36%) (see Table 3). These results suggest that these two compounds play a significant role in inhibiting the synthesis of AFB_1_.

Currently, limited data are available to identify the molecules directly implicated in the inhibition of mycotoxin synthesis. Norton et al. studied how certain anthocyanidins and related flavonoids affected AFB_1_ inhibition and showed that, at a concentration of 83 µg of catechin per milliliter culture medium, these anthocyanidins and flavonoids generated 30% AFB_1_ inhibition [52]. Similarly, a microdilution study by Zhou et al. [53] of how a tea-derived polyphenol mixture affected AFB_1_ synthesis showed that catechin at a concentration of 500 µg/mL of solvent generates 50% AFB_1_ inhibition. Zhou et al. [53] also suggested that using a mixture of polyphenols, including epicatechin, epigallocatechin, and/or gallocatechin, could enhance the inhibition of AFB_1_.

The present results indicate that catechin and epicatechin both inhibit AFB_1_. However, considering the effects of these two compounds when used alone, one would naively expect a total AFB_1_ inhibition of over 80% when the two compounds are used together. This expectation significantly exceeds the inhibition obtained when using both *AM* and *UT* aqueous extracts. This lower efficacy may be attributed to the fact that, in the plant extracts, these two compounds are present as polymers, whereas the standards were tested as monomers. Therefore, the effectiveness of the plant extracts may be strengthened by their depolymerization. Such a drop in efficacy could also result from an inhibition–saturation phenomenon for AFB_1_ if both compounds target the same reaction pathway. Further studies are thus required to determine the exact reaction mechanism by which these molecules impact AFB_1_ synthesis.

The results also indicate that catechin may affect other mycotoxins besides AFB_1_. In fact, a chemical characterization of a methanolic extract of *Aloe vera* gel with high anti-ochratoxin A potential suggested that catechin is a primary contributor to this inhibitory process [54].

Combining these results allows us to propose catechin and epicatechin as compounds with promising anti-AFB_1_ activity (and, more generally, antimycotoxin activity). However, more detailed studies are needed on the reaction mechanism(s) and on possible interactions between catechin and epicatechin.

## 3. Conclusions

The present results demonstrate that aqueous extracts of *AM* and *UT* inhibit AFB_1_ synthesis without affecting the growth of *A. flavus*. The bio-guided fractionation demonstrates the importance of polyphenols, especially condensed tannins, in inhibiting AFB_1_ synthesis. Although *AM* and *UT* are unrelated plants, the results show that catechin and epicatechin are the primary condensed tannins present in aqueous extracts from each plant. The results show that these compounds play an essential role in AFB_1_ inhibition, opening the door to the valorization of certain agricultural by-products rich in condensed tannins. These by-products could be used during or after harvest to protect crops from AFB_1_ contamination. The formulation of such a product is a key point to ensure the correct distribution of the active compounds throughout the grains. Finally, more research is required to determine the precise reaction mechanism(s) of these compounds and to verify that the inhibitory effect is reproduced in other *A. flavus* strains and other aflatoxigenic species.

## 4. Materials and Methods

### 4.1. Chemicals and Reagents

All analytical solvents and chemicals were purchased from Sigma-Aldrich (St Quentin-Fallavier, France). (+)− epicatechin and (−)− catechin were purchased from Extrasynthese (Genay, France). Menthofuran (3,6-dimethyl-4,5,6,7-tetrahydro-1-benzofuran, ≥95%) was purchased from Thermo-Fisher (Strasbourg, France). Ethanol (96%) and sodium carbonate were purchased from VWR International (Fontenay sous Bois, France).

### 4.2. Plant Material

Dried *Annona muricata leaves* and *Uncaria tomentosa bark* were purchased from a local market in Lima, Peru. The samples were ground using a mill with a 2 mm grid and stored until needed at 4 °C in plastic bags.

### 4.3. Preparation of Aqueous Extracts

The aqueous extraction was performed by maceration in a 5 L reactor. Ninety grams of ground material from each plant was combined with 3 L of ultrapure water and stirred for 15 h at room temperature. The mixture was then centrifuged for 15 min at 15,000 rpm (Sigma 6-16 K Centrifuge, Osterode AM Harz, Germany) and filtered under vacuum in a Whatman No. 1 paper (Cytiva). The extracts were subsequently sterilized at 121 °C for 20 min and stored at 4 °C until required.

### 4.4. Fractionation of Extracts

The fractionation method was adapted from Hernandez et al. [15] and involved macroporous adsorption resin amberlite FPX66 (Rohm and Haas, Philadelphia, PA, USA). First, 80 g of resin was preconditioned with 250 mL of ultrapure water (UHQ) stirred by a magnetic stirrer for 10 h. Subsequently, the solution was packed into a cylindrical glass column (inner diameter × length = 3 cm × 30 cm) fitted with a fritted disk. Three hundred mL of each extract was deposited into the column containing the resin. A first elution with 600 mL of ultrapure water (UHQ) was performed to remove sugars and proteins (fraction 1), followed by desorption using 375 mL of 96% ethanol to elute the compounds contained in the resin (fraction 2). The entire fractionation was conducted three times, and the fractions obtained with each solvent were pooled.

### 4.5. PVPP Fractionation

The condensed tannins in the ethanolic fraction (fraction 2) were precipitated using the nonprotein polymer polyvinylpolypyrrolidone (PVPP) to obtain a tannin-free fraction. Briefly, 5 mL of fraction 2 from each extract was combined with 5 mL of distilled water and 500 mg of PVPP. The samples were vortexed for 30 s, then stored at 4 °C for 15 min, and then centrifuged at 4000 rpm (3000 g) for 10 min. The supernatant was collected for subsequent analysis (fraction 2.1).

### 4.6. Characterization of Extracts and Fractions

#### 4.6.1. Dry Matter Content

The DM content was determined by weighing 20 mL of each sample before and after drying in a Memmert oven (Schwabach, Germany) at 100 °C for 24 h. This procedure was performed in triplicate.

#### 4.6.2. Dosage of Total Phenolic Content

The total phenolic content was assessed using the Folin–Ciocalteu method from Singleton and Rossi in a 96-well microplate [55]. The results are expressed in mg of gallic acid equivalent (GAE) per gram of dry extract.

#### 4.6.3. Condensed Tannin Content

The condensed tannin content of each sample was quantified by using the Waterman and Mole method [56]. The following equation was used to calculate the tannin concentration:

Tannin concentration (mg/g) = [0.3866 × (sample absorbance − control absorbance) × dilution factor]/(dry matter)

All tests were performed three times, and the results are expressed in milligrams of condensed tannins per gram of dry matter (DM) ± one standard deviation.

#### 4.6.4. Dosage of Free Radical Scavenging Activity

The antioxidant activity was assessed using the DPPH (2,2-diphenyl-1-picrylhydrazyl) method from Brand-Williams et al. [57]. The radical-scavenging activity was reported as the half-maximal inhibitory concentration (IC50), which corresponds to the concentration of the extract (in mg/L) required to reduce 50% of the DPPH radicals. IC50 was calculated as follows:

$$IC50=0.5-\frac{a}{b}$$

The results are expressed in milligrams of extract per liter (mg/L).

### 4.7. Characterization of Condensed Tannins in the Ethanolic Fraction of AM and UT Extracts

#### 4.7.1. Depolymerization of Condensed Tannins from Fraction 2

In a vial, 500 µL of methanolic solutions of *AM* and *UT* (2 g/L) previously prepared from a dried fraction 2 of both samples was mixed with 50 µL of menthofuran (13.8 mM) and 450 µL of HCl (0.1 M). The vials were sealed and incubated at 30 °C. After 120 min of reaction, the vials were immediately analyzed by UHPLC-DAD-MS (Thermo Scientific, Waltham, MA, USA). Each test was performed in triplicate per sample.

#### 4.7.2. UHPLC-DAD-MS Analyses

The liquid chromatography system included an Ultimate 3000 ultrahigh-performance liquid chromatography (UHPLC) instrument equipped with a photodiode array detector (Thermo Fisher Scientific, Waltham, MA, USA). The column consisted of a Kinetex 2.6 μm EVO C18 100 Å, 150 mm × 4.6 mm (Phenomenex, Torrance, CA, USA) maintained at 40 °C. The flow rate was 1.40 mL/min and the gradient conditions were as follows: solvent A (H_2_O–HCOOH, 999:1, *v*/*v*), solvent B (CH3CN–HCOOH, 999:1, *v*/*v*); 0–7 min, 1% to 60% B (linear gradient); 7–10 min, 60% to 99% B (linear); 10–11 min, 99% B (isocratic); 11–12 min, 99% to 1% B (linear); 12–13 min, 1% B (isocratic). The Ultimate 3000 UHPLC system was coupled online with an amaZon SL Ion-Trap mass spectrometer (Bruker Daltonics, Billerica, MA, USA), operating in positive-ion mode with electrospray ionization. In the source, the nebulizer pressure was set to 44 psi, the dry gas temperature was maintained at 200 °C with a flow rate of 10 L/min, and the capillary voltage was adjusted to 4.5 kV. Mass spectra were acquired in UltraScan mode over an m/z range of 100–1600 with a mass spectrum-acquisition speed of 8100 (*m*/*z*) s^−1^.

#### 4.7.3. Identification of Peaks and Quantification of Products

Peaks 1 and 2 from the UV chromatograms (280 nm) were attributed to (+)-catechin and (−)-epicatechin, respectively, by comparing their associated mass spectra and retention times with those obtained from pure standards. The products resulting from the trapping of extension units by menthofuran (i.e., (epi)catechin-(4 → 5)-menthofuran) are not commercially available. Peak 3 was attributed to catechin-(4 → 5)-menthofuran by comparing its associated mass spectrum and retention time with that obtained by depolymerizing a commercial standard of procyanidin B3 with menthofuran (this reaction yields only catechin and the targeted catechin-(4 → 5)-menthofuran). Peak 4 was attributed to epicatechin-(4 → 5)-menthofuran by using the same method applied to procyanidin B2. In a previous study, the procyanidin depolymerization products obtained using menthofuran were also characterized by NMR [58].

The molar responses of catechin and epicatechin at 280 nm were determined and found to be equal through calibration with their respective commercial standards. This molar response at 280 nm was applied to quantify the corresponding extension units because (epi)catechin-menthofuran has the same molar response at 280 nm as (epi)catechin [58].

### 4.8. Effect of U. tomentosa and A. muricata Extracts and Fractions on Aspergillus flavus Growth and Aflatoxin B1 Synthesis

#### 4.8.1. Fungal Strain and Culture Conditions

*Aspergillus flavus* NRRL 62,477 strain was used for all analyses. First, a spore suspension in 0.05% Tween 80 was prepared from a seven-day-old culture maintained at 27 °C in malt extract agar (Biokar Diagnostics, Allone, France). The concentration of the spore suspension was adjusted to 10⁵ spores/mL. Petri dishes were then prepared by adding 18 mL of malt extract agar medium and 2 mL of the extract or fraction at four concentrations (0.04, 0.08, 0.15, and 0.3 mg DM per milliliter culture medium) diluted in water. Control cultures were prepared by adding 2 mL of distilled water to the culture medium. Finally, 10 μL of the spore suspension was inoculated centrally onto each Petri dish and incubated for 7 days at 27 °C in the dark. After incubation, the colony diameter was measured to assess the fungal growth. Each measurement was performed in triplicate.

For catechin and epicatechin assays, the standards were dissolved in methanol to obtain an initial concentration of 1 mg/mL. Different concentrations were then prepared by dilution in distilled water: 300, 200, and 100 µg/mL for catechin, and 500, 250, and 125 µg/mL for epicatechin. Next, 2 mL of the standard solutions was added to 18 mL of malt extract agar; control cultures were prepared by adding 2 mL of a mixture of distilled water and methanol (3:1, *v*:*v*).

All cultures were incubated 7 days at 27 °C before analysis of AFB_1_ production.

#### 4.8.2. Aflatoxin Extraction and HPLC Quantification

AFB_1_ extraction and quantification was performed as described by El Khoury et al. [5]. Aflatoxin B1 was quantified by using an Ultimate 3000 UHPLC system (Thermo Fisher Scientific) with an EvoC18 column (3 μm, 150 mm × 3.2 mm, Phenomenex) at 27 °C. AFB_1_ was detected with a fluorescence detector at 365 nm excitation and 430 nm emission. Additionally, to confirm the nature of the molecules, the UV spectra were analyzed using a DAD integrated into the system.

We calculated the half-maximal inhibitory concentration to inhibit AFB_1_ (IC50*_AFB_*_1_), which corresponds to the concentration of extract (in mg/L) required to reduce AFB_1_ synthesis by 50%. The equation shown in Section 4.6.4 was used for the calculation.

### 4.9. Statistics

The differences between control and treated samples were analyzed using the Student’s *t*-test. Statistical significance was defined as *p* values less than 0.05. All errors were reported as the standard deviation of the mean. Data analyses were carried out using R studio software (version 1.4.1717).

## Figures and Tables

**Table 1:** Characterization of *AM* and *UT* aqueous extract, aqueous (F1) and ethanolic (F2) fractions after separation on macroporous resin, and subfraction 2.1 (tannin-free fraction) obtained by PVPP precipitation.

Fraction	Dry Matter (g)	Polyphenols Content (mg GAE/g DM) *	Antioxidant Activity IC50_DPPH_ (mg/L) **	Condensed Tannins (mg/g DM)
*AM*	3.91 ± 0.01 ^a^	188 ± 9 ^a^	35 ^a^	137 ± 2 ^a^
F1	1.70 ± 0.02 ^b^	56 ± 5 ^b^	310 ^b^	Nd
F2	1.83 ± 0.04 ^b^	320 ± 1 ^b^	20 ^b^	220 ± 4 ^b^
F2.1	0.21 ± 0.01 ^b^	10 ± 1 ^b^	460 ^b^	2 ± 1 ^b^
*UT*	5.31 ± 0.03 ^a^	226 ± 3 ^a^	15 ^a^	219 ± 5 ^a^
F1	1.92 ± 0.01 ^b^	18 ± 2 ^b^	363 ^b^	Nd
F2	2.24 ± 0.01 ^b^	530 ± 7 ^b^	8 ^b^	502 ± 6 ^b^
F2.1	0.18 ± 0.03 ^b^	6 ± 1 ^b^	>500 ^b^	3 ± 1 ^b^

* DM: dry matter; GAE: gallic acid equivalent; ** Concentration of extract or fraction reducing 50% of DPPH (2,2-Diphenyl-1-picrylhydrazyl), Nd: not detected. For each parameter, significant differences between the initial plant extract and its fractions are indicated by different letters (*p* value < 0.05).

**Table 2:** Primary condensed tannins present in F2 fractions of *AM* and *UT*.

Composition in Condensed Tannins of the Tested Fractions (%*w*/*w*)					
Peak Number	1	2	3	4	
Detected compound	Catechin (terminal units)	Epicatechin (terminal units)	Catechin-Mf * (extension units)	Epicatechin-Mf * (extension units)	Total procyanidin content
Tested fractions					
*AM-F2*	2.8 ± 0.1	1.7 ± 0.1	2.5 ± 0.1	8.9 ± 0.1	15.9
*UT-F2*	1.1 ± 0.1	9.3 ± 0.1	4.3 ± 0.1	16.4 ± 0.2	31.1

* Mf: menthofuran.

**Table 3:** Effect of increasing concentrations of catechin and epicatechin standards on AFB_1_ inhibition.

Standard	Concentration of the Standard (µg/mL of Culture Medium)	Inhibition of AFB_1_ * (%)
Catechin	5.00	28 ± 1
	10.00	37 ± 2
	15.00	45 ± 1
Epicatechin	6.25	19 ± 1
	12.50	27 ± 2
	25.00	36 ± 2

* Results are expressed as the percent of AFB_1_ inhibition with respect to untreated control cultures.

## Data Availability

The data presented in this study are available on request from the corresponding author.
